# Neuroradiological Endovascular Intervention for Diplopia in a Case of Aneurysmal Aberrant Regeneration of the Third Nerve

**DOI:** 10.7759/cureus.1340

**Published:** 2017-06-12

**Authors:** Anupam C.A. Rao, Saumil A. Shah, Benjamin W.C. Sim, Steven T.H. Yun, Neeranjali S. Jain, Yashar Kalani, Ian C. Francis

**Affiliations:** 1 Department of Ophthalmology, Prince of Wales Hospital; 2 Faculty of Medicine, University of New South Wales; 3 Department of Ophthalmology, Sydney Eye Hospital; 4 Division of Neurological Surgery, Barrow Neurological Institute

**Keywords:** posterior communicating artery aneurysm, third nerve palsy, endovascular coiling, aberrant regeneration of third nerve, oculomotor synkinesis, vertical diplopia

## Abstract

Aberrant regeneration of the third nerve occurs as a result of synkinetic ‘miswiring’ of the third nerve following its injury, such as in third cranial nerve palsy due to tumor, trauma, or aneurysm. The case presented is an elderly woman with new vertical diplopia, which led to a diagnosis of a third cranial nerve palsy, thought to be caused by a 5 mm blister aneurysm of the posterior communicating artery. However, neuro-ophthalmological evaluation diagnosed aberrant regeneration of the third nerve, with the cause of her new vertical diplopia being an ipsilateral fourth nerve palsy. The patient underwent endovascular treatment of her aneurysm using stent-assisted coiling. This procedure was complicated by an episode of air embolism, from which the patient made a good recovery. This patient’s presentation demonstrates that the cause of any diplopia must be established, and presents a novel, semi-schematic illustration of aberrant regeneration of the third nerve that should aid clinicians in its recognition.

## Introduction

Aberrant regeneration of the third nerve (Ab3), also known as oculomotor synkinesis, is a phenomenon usually resulting from trauma, tumors, and aneurysms. It usually follows a third nerve palsy (3NP) but may occur as a primary disorder with no preceding nerve dysfunction [1]. The misdirected regeneration of axons leads to a clinical syndrome of unilateral defective adduction, defective vertical gaze, pupillary constriction on attempted adduction or downgaze, and upper eyelid elevation on adduction or downgaze [1].

The case presented is of an elderly woman whose new vertical diplopia led to her aberrant regeneration of the third nerve being diagnosed as a third nerve palsy. Her vertical diplopia was in fact later shown to be due to a new ipsilateral fourth nerve palsy (4NP), rather than an Ab3. The immediate diagnosis and the presence of a small posterior communicating artery (PcomA) blister aneurysm led to neuroradiological endovascular intervention, which in turn caused complications.

The clinical features of an Ab3, highlighting the importance of recognition of this entity, are presented.

## Case presentation

**Figure 1:**
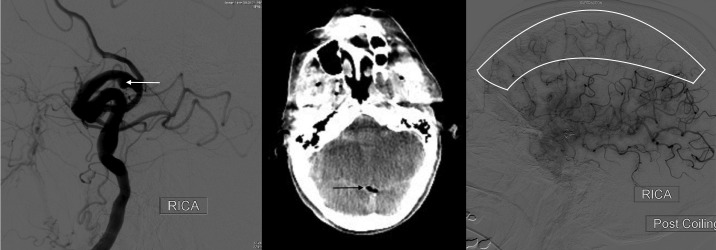
Imaging results. A. Internal carotid artery lateral view computed tomography (CT) angiogram demonstrates a 5 mm x 3 mm blister aneurysm (white arrow) at the origin of the Posterior communicating artery. B. Post-interventional axial CT head demonstrating air (black arrow) in the straight sinus. C. Internal carotid artery lateral view digital subtraction angiogram in the capillary phase demonstrating filling defects (area surrounded by white lines) caused by air embolization in territory of middle cerebral artery.

An 81-year-old healthy, independent lady presented with a 5-day history of vertical diplopia and vertigo. She had no history of smoking, hypertension, or diabetes. Initial outpatient examination revealed limited adduction of the right eye, with intermittent vertical strabismus. She had a sluggish and moderately dilated right pupil, but no ptosis. A diagnosis of an acute right 3NP was made and the patient underwent an immediate computed tomography (CT) angiogram that revealed an ipsilateral 5 mm PcomA blister aneurysm (Figure 1A). She was referred for neurosurgical intervention. Given the presence of the documented '3NP', the patient was scheduled for stent-assisted coiling embolization of the aneurysm.

On admission, the patient demonstrated diminished adduction and depression in adduction of the right eye, which was confirmed collaterally to have persisted for many years. Ipsilateral upper lid retraction and pupillary miosis were present on attempted adduction and depression of the right eye. The velocity of the adducting saccade in the right eye was reduced. The right eye displayed absence of intorsion in conjugate downgaze to the right, confirming an ipsilateral 4th nerve palsy (4NP). The findings, demonstrated by the neuro-ophthalmology team, were diagnostic of Ab3 with an associated ipsilateral 4NP, excluding an acute 3NP. On the basis that the patient had a PcomA aneurysm with a ‘3NP’, she underwent neuroradiological endovascular intervention for the PcomA aneurysm.

The stent-assisted coiling embolization of the aneurysm was successful. However, the procedure was complicated by air emboli (Figure 1B). Widespread cerebral ischaemia was seen on the post-coiling digital subtraction angiogram (Figure 1C). Antiplatelet agents and hyperbaric oxygen therapy were initiated. The patient developed a left supranuclear horizontal conjugate gaze palsy, a left facial paresis, a left hemiparesis, and subsequent complex partial seizures with oral automatisms.

At review 10 months later, the patient had made a good recovery and was functionally independent. She continues to have ocular findings consistent with Ab3.

## Discussion

It is recognized that 34–56% [1] of third nerve palsies are associated with an expanding PcomA aneurysm. Ab3 is a relatively common sequela of a 3NP, occurring in 15% of patients [2-3]. Ab3 suggests a chronic process, as the nerve attempts to repair the damage caused by an injury sustained several weeks or more prior to diagnosis. Therefore, Ab3 points to a long-standing process rather than an acute abnormality. When Ab3 develops following a third nerve palsy, the literature indicates that it occurs more than 36 days afterward [3]. However, it can occur up to 39 years later [3]. This time course implies a gradual process and not an acute alteration in the status of the aneurysm. The finding of an acute 3NP associated with an aneurysm of the PComA is suggestive of hemodynamic alteration in the aneurysm, and warrants immediate intervention. By contrast, the finding of Ab3 indicates a chronic process which may not require intervention.

The decision-making process regarding small intracranial aneurysms can be challenging. The International Study of Unruptured Intracranial Aneurysms (ISUIA) suggests that PcomA aneurysms, although in the anterior circulation, have a more favourable natural history, closely resembling those in the vertebrobasilar circulation [4]. According to ISUIA, PcomA aneurysms of <7 mm have a favourable natural history, harbouring a 2.5% risk of rupture over five years [4]. However, even these aneurysms, which have a minimal likelihood of rupture, will likely present with subarachnoid haemorrhage. This patient did not have a subarachnoid haemorrhage, suggesting that the risk of rupture was even lower than might otherwise be expected [4].

**Figure 2:**
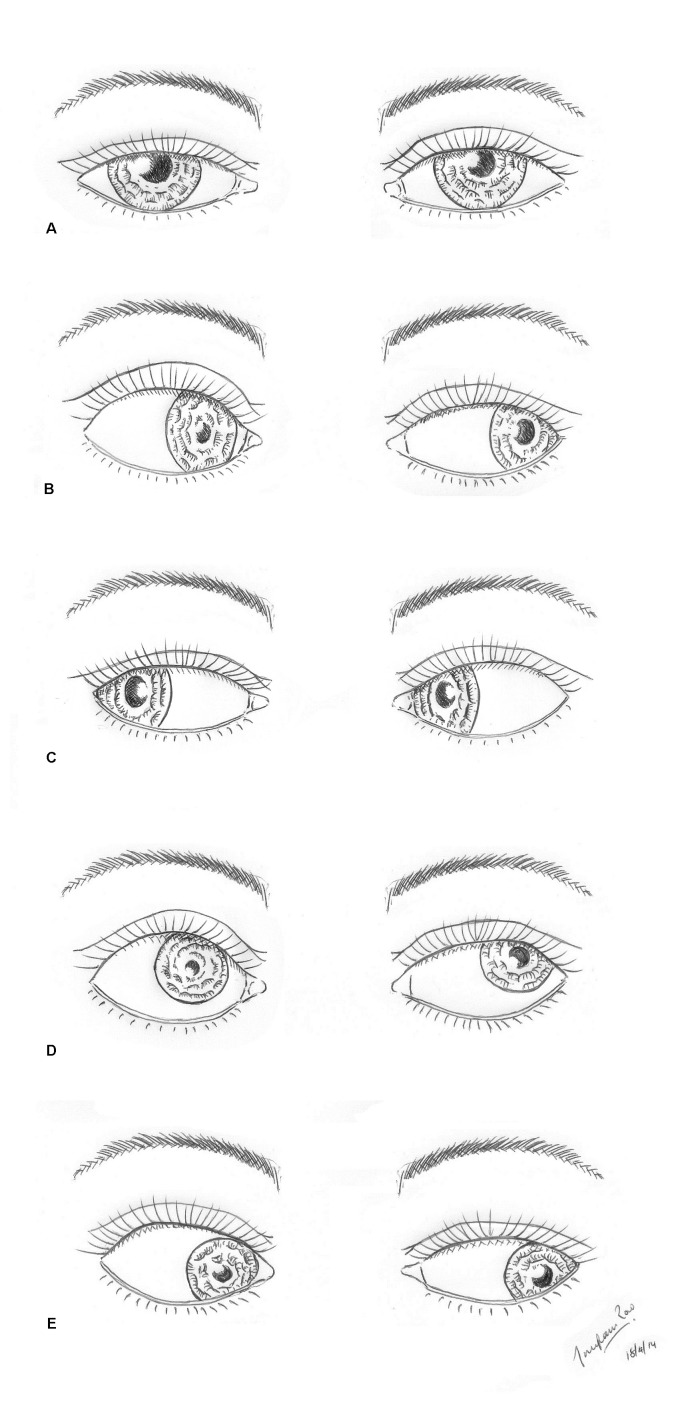
Semi-schematic representation of aberrant regeneration of the right third nerve (Ab3). A. Patient looking in primary position. Note mild right upper lid ptosis and slightly dilated right pupil. B. Patient looking to the left. Note that the right pupil constricts on attempted adduction. The right upper lid demonstrates prominent elevation. The adducting saccadic velocity of the right eye is reduced. C. Patient is looking to the right. Note that right pupil is again slightly dilated, the same size as it was in primary position (as in Figure 2A). The right upper lid again demonstrates mild ptosis. D. Patient is looking up and to the left. Note the right upper lid elevates more than the left upper lid, and the right pupil constricts. E. Patient is looking down and to the left. Note the right pupil constricts (as in Figure 2D). The right upper lid demonstrates retraction.

This case highlights the importance of diagnosing Ab3 in patients with a suspected 3NP. Ab3 is caused by an aberrant, synkinetic ‘miswiring’ of the regenerated nerves [2-3, 5]. Ab3 occurs after non-ischaemic 3NPs, and is most commonly caused by craniocephalic trauma (65%), or intracranial aneurysms (13%), tumors (8%), and surgery (8%) [5]. Ab3 presents with the tetrad of unilateral defective adduction, defective vertical gaze, pupillary constriction on attempted adduction or downgaze, and upper eyelid elevation (lid retraction) on adduction or downgaze [3]. As a diagnostic aid, Figure 2 demonstrates the features of Ab3 in a semi-schematic, stepwise neurological examination.

Since this patient was recognized to have Ab3 within five days of onset of her acute vertical diplopia, her PcomA aneurysm could have been reliably excluded as the cause of the diplopia. Thus her vertical diplopia resulted from her ipsilateral 4NP. Furthermore, her age, the characteristics of the aneurysm, including its size and ‘blister’ characteristics, the presence of the Ab3, and the aetiological 4NP should have encouraged a non-interventional approach to her diplopia [4].

## Conclusions

This case highlights the importance of detailed ocular examination and recognition of an Ab3 as a cause of diplopia in a patient harbouring an intracranial aneurysm. Whereas the finding of an acute 3NP associated with a PComA aneurysm requires prompt intervention due to the risk of rapid aneurysm progression, this patient was found to have an Ab3, indicating a chronic process. Given the characteristics of the aneurysm, the patient’s age and the fact that there was no evidence of an acute 3NP, conservative management would have been more appropriate than neuroradiological intervention.
